# Momelotinib for myelofibrosis: our 14 years of experience with 100 clinical trial patients and recent FDA approval

**DOI:** 10.1038/s41408-024-01029-3

**Published:** 2024-03-18

**Authors:** Ayalew Tefferi, Animesh Pardanani

**Affiliations:** Division of Hematology, Department of Medicine, Mayo Clinic, Rochester, MN USA

**Keywords:** Myeloproliferative disease, Myeloproliferative disease

Momelotinib is an ATP-competitive small molecule inhibitor of Janus kinase proteins (JAKi), including JAK1, JAK2, JAK3, and TYK2; its other clinically relevant targets include activin A receptor type 1 (ACVR1), also known as activin receptor like kinase 2 (ALK2) [1]. Momelotinib was recently approved (September 15, 2023) for use in anemic patients with high/intermediate risk myelofibrosis (MF), including primary (PMF) [2] and secondary variants, the latter emerging from antecedent polycythemia vera (post-PV) [3] or essential thrombocythemia (post-ET) [4]. All three MF variants belong to the broader category of myeloproliferative neoplasms (MPNs), which are characterized by the presence of JAK-STAT activating mutations (*JAK2*, *CALR* or *MPL*) and predominantly megakaryocytic myeloproliferation with variable degrees of bone marrow fibrosis [5]. Patients with MF face premature death with 10-year survival estimates ranging from >80% in very low-risk diseases to <5% in very high-risk diseases [6]. In addition, the clinical course of the disease in MF is complicated by progressive anemia, extramedullary hematopoiesis with marked splenomegaly and hepatomegaly, constitutional symptoms, and cachexia. Causes of death in MF include disease transformation into acute myeloid leukemia [7].

Treatment in MF aspires to prolong life, primarily, and improve quality-of-life (QoL), secondarily. The latter is compromised by severe anemia, marked splenomegaly, profound constitutional symptoms, and progressive cachexia [2]. Drug therapy in MF, including the use of JAKi, has yet to succeed in securing long-term survival, which is currently accomplished only by allogeneic hematopoietic stem cell transplantation (AHSCT) [8]. A recent study of over 4000 patients with MF receiving AHSCT revealed 3-year survival, relapse, and non-relapse mortality rates of 58%, 22%, and 29%, respectively [9]. More importantly, the particular study disclosed a recent shift in the distribution of recipient age and donor source towards older patients and matched unrelated, respectively. For the individual patient, it is important to justify the risk of transplant-related mortality and morbidity, through contemporary molecular prognostication systems [6]. Non-transplant treatment options in MF are mostly palliative with choices made based on treatment indications. Accordingly, anemia is often managed with red blood cell (RBC) transfusions or drug therapy with erythropoiesis stimulating agents (ESAs), androgen preparations, prednisone, danazol, and immunomodulatory drugs (IMiDs; thalidomide, lenalidomide) [2]. Prior to the advent of JAKi, MF-associated splenomegaly and constitutional symptoms were managed mostly with hydroxyurea [10] and in resistant cases with splenectomy [11] or splenic irradiation [12].

The discovery of *JAK2*V617F in 2005 [13] and subsequently *MPL* [14, 15] and *CALR* [16] mutations, led to the development of several JAKi, with the objective to target constitutive JAK-STAT activation, which is believed to be the primary driver of disease phenotype in MF, including splenomegaly and constitutional symptoms [17]. Currently available JAKi are not specific to mutation-specific JAK-STAT activation [18] but their non-specific inhibition of JAK2 and JAK1 resulted in broad suppression of inflammatory cytokines and myeloproliferation with favorable effects on constitutional symptoms and splenomegaly [2]. The demonstration of benefit in QoL, has so far allowed FDA approval of four JAKi: ruxolitinib (November 16, 2011), fedratinib (August 16, 2019), pacritinib (February 28, 2022), and most recently momelotinib (September 15, 2023). None of these JAKi induce morphologic, cytogenetic or molecular remissions and their value is limited to control of splenomegaly and symptoms. In addition, momelotinib has been uniquely identified for its erythropoietic effect, believed to result from its inhibition of ACVR1(ALK2) [19].

Our story with momelotinib started in 2009 with a preclinical study report where we showed IC50 inhibition of <100 nM for JAK1, JAK2, CDK2/cyclin A, JNK1, ROCK2, TBK1, PKD3, and PRKD1 and inhibition of (i) proliferation of *JAK2*V617F harboring HEL and Ba/F3 cell lines, (ii) phosphorylation of STAT-5 and STAT-3 in HEL cells, and (iii) in vitro erythroid colony formation in PV patients [20]. Subsequently, our group led the first in human phase-1/2 study with momelotinib (NCT00935987) in high/intermediate-risk MF, serially published between 2013 and 2023 [21–29] and recently reviewed [1]. A total of 166 patients (143 JAKi-naïve) were enrolled in the study between November 2009 and august 2011 with 165 having received at least one dose of the drug and had at least one baseline efficacy evaluation; drug doses ranged between 100 and 400 mg once-daily while the dose confirmation phase utilized 150 or 300 mg once-daily [27]. The study included 100 patients from the Mayo Clinic with our first patient enrolled on 11/20/2009 and our last on 3/24/2011.

In 2013, we published the very first report on the efficacy and toxicity of momelotinib in the first 60 patients from the above mentioned phase-1/2 study [21]; treatment response according to the international working group (IWG-MRT) criteria [30] included 0% complete remission, 2% partial remission, 57% clinical improvement, 45% anemia response, 53% resolution of transfusion need, and 42% clinically-vetted spleen response [21]; grade 3/4 adverse events considered at least possibly related to momelotinib included thrombocytopenia (32%), increased AST/ALT (3.3%/3.3%), increased lipase (5%), and headache (3.3%); grade 1/2 treatment-related adverse events included thrombocytopenia (27%), nausea (18.3%), diarrhea (13.3%), increased AST/ALT (13.3%/11.7%), increased bilirubin (11.7%), increased lipase (5%), dizziness (25%), peripheral neuropathy (26.7%), headache (13.3%), and flushing (11.7%); comparison of patients receiving 150 mg vs. 300 mg daily dose suggested higher frequency of peripheral neuropathy (33.3% vs. 14.3%) and diarrhea (22.2% vs. 4.8%) with the higher dose [21].

In 2015, we reported detailed account of momelotinib-associated neuropathy among Mayo Clinic patients with documentation of treatment-emergent peripheral neuropathy in 44 (44%) out of 100 treated patients [28]; median time of onset was 32 weeks; improvement after drug dose reduction or discontinuation was documented in 2 patients [28]. Also in 2015, we reported significant associations between spleen response and the presence of *CALR* and absence of *ASXL1* mutation while resolution of RBC transfusion need was favorably impacted by intermediate vs. high risk disease, normal vs. abnormal karyotype, and platelet count <100 × 10(9)/L vs. higher level [29]. In 2018, we reported the long-term results of all 166 patients enrolled in the original phase-1/2 clinical trial [27]; anemia response was 54% and spleen 40% while adverse events included grade-3/4 thrombocytopenia (34%) and neutropenia (8%), grade-1/2 diarrhea (48%), nausea (39%), vomiting (24%), dizziness (40%), peripheral neuropathy (30%), and first-dose effects of flushing, hypotension, dizziness and nausea (11%); in addition, increases in liver function tests and pancreatic enzymes were documented in 15–18% and 11–13%, respectively [27].

Between 2018 and 2023, we reported more mature data in terms of overall survival as well as predictors of treatment response to momelotinib, from the above-described phase-1/2 study [22–24, 26]. In a retrospective comparison between 79 JAKi-naïve patients treated with momelotinib and 50 patients treated with ruxolitinib in a separate clinical trial (NCT00509899) [24], median survivals from initiation of study drug were 3.5 years (10-year survival 20%) for momelotinib and 4.0 years (10-year survival 23%) for ruxolitinib (*p* = 0.32) [24]; however, drug retention was superior for momelotinib, compared to ruxolitinib, with 3-year drug discontinuation rate of 68% vs 88% (*p* < 0.01). A separate publication of 183 Mayo Clinic patients with JAKi-naïve high/intermediate risk MF enrolled in consecutive phase 1/2 JAKi clinical trials that included momelotinib (*N* = 79), ruxolitinib (*N* = 50), fedratinib (*N* = 23), and BMS-911543 (*N* = 31), the 10-year survival rate for all 183 patients was 16% and not significantly different across the four drug cohorts (*p* = 0.33) [22]; multivariable analysis identified age >65 years, absence of type 1/like *CALR* mutation, baseline transfusion need, and presence of *ASXL1*/*SRSF2* mutation as risk factors for survival. In addition, spleen and anemia responses were independently associated with improved short-term survival while long-term survival was secured only by AHSCT (10-year survival rate 45% vs 19% in non-transplanted patients; *p* < 0.01) [22].

In another analysis of 72 Mayo Clinic patients who were JAKi-naïve and anemic prior to treatment with momelotinib [23], 44% experienced anemia response (median response duration ∼20 months; range 3–81); spleen and symptom responses were documented in 45% and 44% of evaluable patients, respectively. In multivariable analysis, predictors of anemia response included post-ET MF (83% vs 37%) and serum ferritin level of <55 mcg/L (89% vs 38%); among all 72 study patients, treatment was discontinued in 93% after median treatment duration of 20 months. Post-momelotinib median survival was 3.2 years with 5 and 10-year survival rates at 31% and 19%, respectively. In multivariable analysis, survival was positively affected by anemia response (median 3.8 vs 2.8 years), presence of type 1/like *CALR* mutation (median 11 vs 3 years), and absence of *ASXL1* or *SRSF2* mutation (median 3.7 vs 2.9 years). The favorable impact of anemia response on survival was also confirmed in transfusion-dependent patients (median 3.7 vs 1.9 years: 10-year survival 8% vs 0%). In our most recent report that was presented at the 2023 annual American Society of Hematology meeting, we found *CALR* type-1/like mutation to be the most prominent favorable risk factor for both overall and drug survival [31], as was also observed by others [32].

Our above-elaborated observations on momelotinib therapy in MF have since been confirmed by multiple phase-2 and phase-3 studies (recently reviewed by Tefferi et al.) [1, 33–35], leading to its recent FDA approval. Although this is welcome news to anemic patients with MF, a number of provisions are in order: (i) the drug is still another JAKi with potential to cause a number of side effects including immune suppression, which requires due diligence in monitoring for opportunistic infections and pre-treatment documentation of routine vaccinations, including for COVID and Herpes Zoster, (ii) the potential for drug-associated peripheral neuropathy must be disclosed to patients and clinical monitoring ensued, and (iii) indiscriminate use in anemic patients with MF who are otherwise not compromised by marked splenomegaly or constitutional symptoms is discouraged since safer and less expensive alternative therapy might be available for such patients. On the other hand, combining momelotinib with other erythropoietic or cytoreductive agents is a promising prospect for enhancing its palliative value in MF. Unfortunately, as has been the case with other JAKi, momelotinib has not been shown to reverse morphological or molecular features of the disease in MF and is unlikely to modify its natural history.

## References

[1] Tefferi A, Pardanani A, Gangat N (2023). Momelotinib (JAK1/JAK2/ACVR1 inhibitor): mechanism of action, clinical trial reports, and therapeutic prospects beyond myelofibrosis. Haematologica.

[2] Tefferi A (2023). Primary myelofibrosis: 2023 update on diagnosis, risk-stratification, and management. Am J Hematol.

[3] Tefferi A, Barbui T (2023). Polycythemia vera: 2024 update on diagnosis, risk-stratification, and management. Am J Hematol.

[4] Tefferi A, Pardanani A (2019). Essential thrombocythemia. N. Engl J Med.

[5] Arber DA, Orazi A, Hasserjian RP, Borowitz MJ, Calvo KR, Kvasnicka HM (2022). International consensus classification of myeloid neoplasms and acute leukemias: integrating morphologic, clinical, and genomic data. Blood.

[6] Tefferi A, Guglielmelli P, Lasho TL, Gangat N, Ketterling RP, Pardanani A (2018). MIPSS70+ version 2.0: mutation and Karyotype-enhanced international prognostic scoring system for primary myelofibrosis. J Clin Oncol.

[7] Tefferi A, Alkhateeb H, Gangat N (2023). Blast phase myeloproliferative neoplasm: contemporary review and 2024 treatment algorithm. Blood Cancer J.

[8] Ali H, Bacigalupo A (2021). 2021 update on allogeneic hematopoietic stem cell transplant for myelofibrosis: a review of current data and applications on risk stratification and management. Am J Hematol.

[9] McLornan D, Eikema DJ, Czerw T, Kroger N, Koster L, Reinhardt HC (2021). Trends in allogeneic haematopoietic cell transplantation for myelofibrosis in Europe between 1995 and 2018: a CMWP of EBMT retrospective analysis. Bone Marrow Transpl.

[10] Sirhan S, Lasho TL, Hanson CA, Mesa RA, Pardanani A, Tefferi A (2008). The presence of JAK2V617F in primary myelofibrosis or its allele burden in polycythemia vera predicts chemosensitivity to hydroxyurea. Am J Hematol.

[11] Tefferi A, Mesa RA, Nagorney DM, Schroeder G, Silverstein MN (2000). Splenectomy in myelofibrosis with myeloid metaplasia: a single-institution experience with 223 patients. Blood.

[12] Elliott MA, Chen MG, Silverstein MN, Tefferi A (1998). Splenic irradiation for symptomatic splenomegaly associated with myelofibrosis with myeloid metaplasia. Br J Haematol.

[13] James C, Ugo V, Le Couedic JP, Staerk J, Delhommeau F, Lacout C (2005). A unique clonal JAK2 mutation leading to constitutive signalling causes polycythaemia vera. Nature.

[14] Pardanani AD, Levine RL, Lasho T, Pikman Y, Mesa RA, Wadleigh M (2006). MPL515 mutations in myeloproliferative and other myeloid disorders: a study of 1182 patients. Blood.

[15] Pikman Y, Lee BH, Mercher T, McDowell E, Ebert BL, Gozo M (2006). MPLW515L is a novel somatic activating mutation in myelofibrosis with myeloid metaplasia. PLoS Med.

[16] Klampfl T, Gisslinger H, Harutyunyan AS, Nivarthi H, Rumi E, Milosevic JD (2013). Somatic mutations of calreticulin in myeloproliferative neoplasms. N. Engl J Med.

[17] Vainchenker W, Leroy E, Gilles L, Marty C, Plo I, Constantinescu SN (2018). JAK inhibitors for the treatment of myeloproliferative neoplasms and other disorders. F1000Res.

[18] Tefferi A, Gangat N, Pardanani A, Crispino JD (2022). Myelofibrosis: genetic characteristics and the emerging therapeutic landscape. Cancer Res.

[19] Oh ST, Talpaz M, Gerds AT, Gupta V, Verstovsek S, Mesa R (2020). ACVR1/JAK1/JAK2 inhibitor momelotinib reverses transfusion dependency and suppresses hepcidin in myelofibrosis phase 2 trial. Blood Adv.

[20] Pardanani A, Lasho T, Smith G, Burns CJ, Fantino E, Tefferi A (2009). CYT387, a selective JAK1/JAK2 inhibitor: in vitro assessment of kinase selectivity and preclinical studies using cell lines and primary cells from polycythemia vera patients. Leukemia.

[21] Pardanani A, Laborde RR, Lasho TL, Finke C, Begna K, Al-Kali A (2013). Safety and efficacy of CYT387, a JAK1 and JAK2 inhibitor, in myelofibrosis. Leukemia.

[22] Gangat N, Begna KH, Al-Kali A, Hogan W, Litzow M, Pardanani A (2023). Determinants of survival and retrospective comparisons of 183 clinical trial patients with myelofibrosis treated with momelotinib, ruxolitinib, fedratinib or BMS- 911543 JAK2 inhibitor. Blood Cancer J.

[23] Gangat N, Begna KH, Al-Kali A, Hogan W, Litzow M, Pardanani A (2023). Predictors of anemia response to momelotinib therapy in myelofibrosis and impact on survival. Am J Hematol.

[24] Tefferi A, Pardanani A, Begna KH, Al-Kali A, Hogan WJ, Litzow MR (2022). Momelotinib for myelofibrosis: 12-year survival data and retrospective comparison to ruxolitinib. Am J Hematol.

[25] Tefferi A, Gangat N, Pardanani A (2022). Jaktinib (JAK1/2 inhibitor): a momelotinib derivative with similar activity and optimized dosing schedule. Am J Hematol.

[26] Tefferi A, Barraco D, Lasho TL, Shah S, Begna KH, Al-Kali A (2018). Momelotinib therapy for myelofibrosis: a 7-year follow-up. Blood Cancer J.

[27] Pardanani A, Gotlib J, Roberts AW, Wadleigh M, Sirhan S, Kawashima J (2018). Long-term efficacy and safety of momelotinib, a JAK1 and JAK2 inhibitor, for the treatment of myelofibrosis. Leukemia.

[28] Abdelrahman RA, Begna KH, Al-Kali A, Hogan WJ, Litzow MR, Pardanani A (2015). Momelotinib treatment-emergent neuropathy: prevalence, risk factors and outcome in 100 patients with myelofibrosis. Br J Haematol.

[29] Pardanani A, Abdelrahman RA, Finke C, Lasho TT, Begna KH, Al-Kali A (2015). Genetic determinants of response and survival in momelotinib-treated patients with myelofibrosis. Leukemia.

[30] Tefferi A, Cervantes F, Mesa R, Passamonti F, Verstovsek S, Vannucchi AM (2013). Revised response criteria for myelofibrosis: International Working Group-Myeloproliferative Neoplasms Research and Treatment (IWG-MRT) and European LeukemiaNet (ELN) consensus report. Blood.

[31] Tefferi A, Pardanani A, Begna K, Al-Kali A Type 1/like Calr mutation in momelotinib-treated patients with myelofibrosis is the most prominent predictor of drug survival and longevity without transplant. Blood Cancer J. 2023; in press. 10.1038/s41408-024-01028-4 PMC10951334 38503764

[32] Mesa R, Harrison C, Oh ST, Gerds AT, Gupta V, Catalano J (2022). Overall survival in the SIMPLIFY-1 and SIMPLIFY-2 phase 3 trials of momelotinib in patients with myelofibrosis. Leukemia.

[33] Mesa RA, Kiladjian JJ, Catalano JV, Devos T, Egyed M, Hellmann A (2017). SIMPLIFY-1: a phase III randomized trial of Momelotinib versus ruxolitinib in Janus kinase inhibitor-naive patients with myelofibrosis. J Clin Oncol.

[34] Harrison CN, Vannucchi AM, Platzbecker U, Cervantes F, Gupta V, Lavie D (2018). Momelotinib versus best available therapy in patients with myelofibrosis previously treated with ruxolitinib (SIMPLIFY 2): a randomised, open-label, phase 3 trial. Lancet Haematol.

[35] Verstovsek S, Gerds AT, Vannucchi AM, Al-Ali HK, Lavie D, Kuykendall AT (2023). Momelotinib versus danazol in symptomatic patients with anaemia and myelofibrosis (MOMENTUM): results from an international, double-blind, randomised, controlled, phase 3 study. Lancet.

